# Comparing the detectability of pulmonary nodules on two ultra-high resolution CT scanners: a preliminary phantom study

**DOI:** 10.1007/s00330-026-12582-9

**Published:** 2026-04-29

**Authors:** Joël Greffier, Cécile Salvat, Maxime Pastor, Nicolas Villani, Valérie Bousson, Ariane Vallot, Salim Si-Mohamed, Fabien de Oliveira, Jean-Paul Beregi, Djamel Dabli, Lama Hadid-Beurrier

**Affiliations:** 1 IMAGINE UR UM 103, Montpellier University, Department of Medical Imaging, Nîmes University Hospital, Nîmes, France; 2 Service de Radioprotection et de physique médicale, Hôpital Lariboisière, APHP. Nord-Université de Paris, Paris, France; 3 Guilloz Imaging Department, University Hospital Center of Nancy, Nancy, France; 4 Service d’Imagerie OstéoArticulaire, Viscérale et Vasculaire, Hôpital Lariboisière Fernand Widal, APHP.Nord-Université de Paris, Paris, France; 5 Université de Lyon, INSA-Lyon, Université Claude Bernard Lyon 1, UJM-Saint Etienne, CNRS, INSERM, Villeurbanne, France; 6 Department of Radiology, Hôpital Louis Pradel, Hospices Civils de Lyon, Bron, France

**Keywords:** Multidetector computed tomography, Thorax, Image enhancement, Phantoms, Imaging multiple pulmonary nodules

## Abstract

**Objectives:**

To objectively and subjectively compare the detectability of pulmonary nodules on an ultra-high-resolution CT (UHR-CT) scanner equipped with energy-integrating detectors and a photon-counting CT (PCCT) in UHR mode.

**Materials and methods:**

An image quality phantom and an anthropomorphic phantom were scanned on a UHR-CT and a PCCT in UHR mode at 7.5/2.5/0.4 mGy. The parameters typically used for UHR chest CT scans were selected. Noise power spectrum and task-based transfer function were computed to assess noise-magnitude, noise texture (*f*_av_), and spatial resolution (*f*_50_), respectively. Detectability indices (*d*’) were computed to model the detection of three chest nodules. Five radiologists subjectively evaluated their confidence in detecting these nodules available on the anthropomorphic phantom.

**Results:**

For PCCT, noise magnitude increased, and *f*_av_ values were similar as the dose decreased. For UHR-CT, noise-magnitude decreased, and *f*_av_ values decreased as the dose decreased. For both inserts and CT systems, *f*_50_ values decreased as the dose decreased. For both inserts and at all dose levels, *f*_50_ values were higher with PCCT than with UHR-CT. *d*’ values increased as the dose increased with PCCT, and the opposite was found with UHR-CT. Confidence in nodule detection was considered clinically sufficient at all dose levels for high-contrast solid nodules at 7.5 and 2.5 mGy for subsolid nodules, and only for PCCT at 7.5 mGy for low-contrast nodules.

**Conclusion:**

Under UHR conditions, pulmonary nodule detectability strongly depends on the dose level and reconstruction parameters. High-contrast nodule detection can be performed at all dose levels with both CT systems, but reconstruction strategies must be optimized for low-contrast nodule detection.

**Key Points:**

- *Do the performance of UHR-CT and PCCT scanners improve the detectability of pulmonary nodules*?
- *PCCT showed dose-invariant noise texture and higher effective spatial resolution, whereas UHR-CT relied on dose-dependent noise suppression, producing smoother images and reduced spatial frequency*.
- *High-contrast nodule detection can be performed at all dose levels with both CT systems, particularly at ultra-low dose levels, but reconstruction strategies must be optimized for low-contrast nodule detection*.

**Graphical Abstract:**

**Figure:**
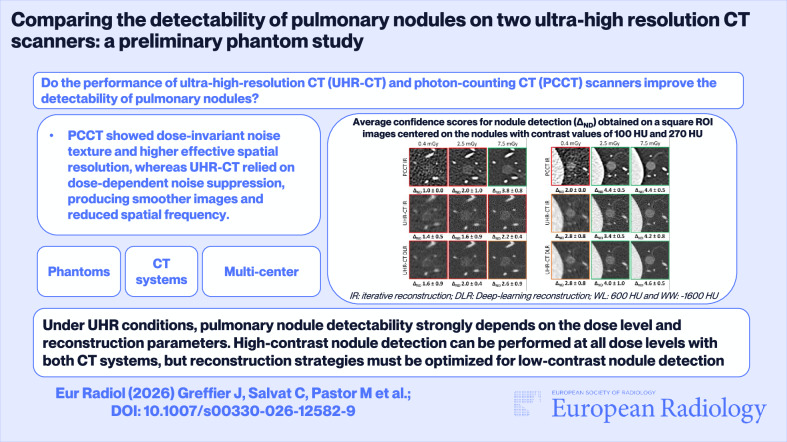

## Introduction

Chest computed tomography (CT) is the standard means of imaging for evaluating pulmonary diseases [1–5] and screening for lung cancer, the leading cause of cancer-related mortality worldwide [6, 7]. Large, randomized trials have demonstrated that low-dose (LD) CT screening significantly reduces lung cancer mortality, and organized screening programs have been implemented in Europe and worldwide [8–10]. Accordingly, the European Society of Thoracic Imaging recommends LD and ultra-low-dose (ULD) CT protocols, with radiation exposure adapted to the patient’s morphology, to minimize cumulative doses, to optimize the risk-benefit ratio whilst preserving diagnostic performance for pulmonary nodule detection and characterization [7, 11–13].

Although dose reduction strategies are essential, they are often associated with image quality degradation, mainly due to greater image noise and potential loss of spatial resolution. These limitations can impair the detection of small pulmonary nodules, particularly in ULD acquisitions [11]. To overcome the spatial resolution challenge, ultra-high-resolution (UHR) CT systems based on smaller energy-integrating detectors (EIDs) and extended reconstruction matrices have been developed. These advances allow reconstruction with smaller voxels and improve visualization of fine pulmonary structures [14, 15]. However, gains in spatial resolution are frequently achieved at the expense of greater image noise or higher radiation doses [16]. To address this trade-off, deep learning-based reconstruction (DLR) algorithms have been introduced. Compared with conventional iterative reconstruction (IR) techniques, DLR provides more effective noise reduction whilst preserving anatomical detail and improving lung lesion detectability at LD levels [17–21].

Another major innovation is the development of photon-counting CT (PCCT) scanners equipped with photon-counting detectors (PCDs). The detectors’ characteristics improve dose efficiency, eliminate electronic noise, and make it possible to use smaller detection pixels (from 0.15 to 0.4 mm) in UHR mode [22]. These advantages, combined with the use of extended reconstruction matrices, result in greater spatial resolution and better noise texture than with conventional EID-CT [22]. These intrinsic properties are particularly relevant for UHR chest imaging, where accurate representation of small pulmonary nodules across a wide range of contrasts is essential [23]. Previous phantom and clinical studies have shown that PCCT improves image quality and lesion detectability in LD and ULD chest CT examinations [13, 23–28]. Although several studies have compared PCCT and EID-CT systems, direct comparisons between state-of-the-art UHR-PCCT and UHR-EID-CT scanners focusing on pulmonary nodule detection remain limited.

The purpose of this study was to compare the detectability of small pulmonary nodules on a PCCT system in UHR mode and on a UHR-EID-CT system at clinically relevant dose levels, including LD and ULD CT acquisitions.

## Materials and methods

### Phantoms

The 26-cm diameter section of the Mercury v4.0 phantom (Sun Nuclear) was used to perform a task-based image quality assessment. This section was chosen to be as close as possible to the body mass index (BMI) of patients undergoing chest CT acquisitions (BMI 20 kg/m²).

The Multipurpose Chest Phantom N1 “LUNGMAN” (Kyoto Kagaku) was used for the subjective image quality assessment and chest nodule detection. In this phantom, 15 simulated spherical pulmonary nodules can be placed inside the phantom: three varieties of Hounsfield unit (HU) values [100 HU (polyurethane, SZ50 and hydroxyapatite), −630 HU (urethane foam) and −800 HU (urethane foam)] and five sizes for each type (diameters of 3, 5, 8, 10 and 12 mm). In the present study, three spherical pulmonary nodules with a diameter of 5 mm, one for each HU value, were positioned in different parts of the phantom [29]. The choice of nodule positioning and diameter was made in consultation with expert radiologists from our institutions.

### CT systems and parameters

Acquisitions were performed on the UHR-CT Aquilion Precision system (Canon Medical Systems) equipped with EIDs, and the PCCT NAEOTOM Alpha.Peak system (Siemens Healthineers) equipped with PCDs. For PCCT, all acquisitions were made using a single X-ray tube and UHR mode (beam collimation of 120 × 0.2 mm). For UHR-CT, the physical beam collimation used was 160 × 0.25 mm. For both CT systems, a tube voltage of 120 kVp was used, and tube current values were set to obtain three volume CT dose levels (CTDI_vol_) of 7.5, 2.5, and 0.4 mGy. The first dose level corresponded to one of our national diagnostic reference levels for the chest (a target value for optimization; 50th percentile), whereas the other two corresponded to the standard (LD) and ultra-low dose (ULD) levels, respectively, used in clinical routine at our institution. For each dose level, ten acquisitions were made on an image quality phantom and one on the anthropomorphic phantom. Table 1 summarizes the parameters used for each CT system.

**Table 1:** Acquisition and reconstruction parameters used on the two CT systems

CT system	PCCT	UHR-CT
Model	NAEOTOM Alpha.Peak	Aquilion Precision
Manufacturer	Siemens Healthineers	Canon Medical Systems
Detector type	Photon-counting	Energy-integrating
Tube voltage (kVp)	120	120
Beam collimation (mm)	120 × 0.2	160 × 0.25
Pitch factor	1.2	1.188
Rotation time (s/rot)	0.25	0.35
Displayed CTDI_vol_ (mGy)	0.40/2.49/7.54	0.40/2.50/7.50
Tube current (mA)	5/31/94	20/120/350
Slice thickness (overlapped)	0.2 mm (0.2 mm)	0.25 mm (0.25 mm)
Reconstruction algorithm(s) used (type)	QIR (IR)	AIDR 3D (IR) AiCE (DLR)
Level(s) used	3	Standard Standard
Kernel(s) used	Bl60	FC51 Lung
Matrix size (pixels)	1024 × 1024	1024 × 1024

*CT* computed tomography, *CTDI*_vol_ volume CT dose index, *DLR* deep-learning image reconstruction, *IR* Iterative reconstruction, *PCCT* photon-counting CT, *UHR* ultra-high resolution

To facilitate comparison of the results, the most frequently used reconstruction parameters for chest CT images on each CT system were used. For PCCT, raw data were reconstructed using Level 3 of the Quantum Iterative Reconstruction (QIR) algorithm, the “Bl60” lung kernel, and a minimum slice thickness of 0.2 mm (0.2 mm overlapped). For UHR-CT, raw data were reconstructed using kernel FC51 for the AIDR 3D IR algorithm and the Lung kernel for the AiCE DLR algorithm. For both algorithms, the Standard level and the 0.25 mm minimum slice thickness (0.25 mm overlapped) were used. For both CT systems, a matrix size of 1024² pixels and fields of view of 280 mm and 350 mm were selected for the image quality phantom and for the anthropomorphic phantom, respectively.

### Task-based image quality assessment

**Fig. 1:**
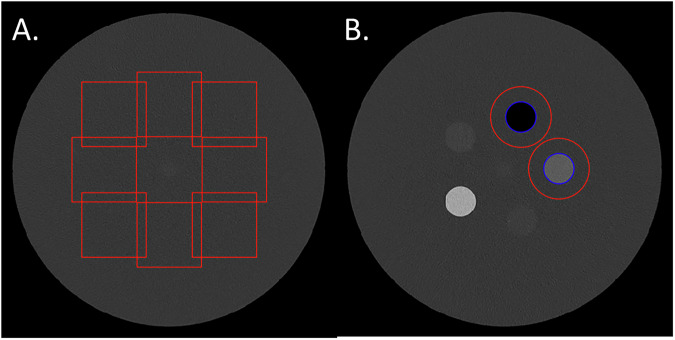
Photographs of the phantom used in this study. **A** Eight square regions of interest (ROIs) were used to assess the noise power spectrum (NPS). **B** Circular ROIs were used to compute the task-based transfer function (TTF) on air and iodine at 10 mg/mL inserts

A task-based image quality assessment was performed using iQMetrix-CT software v1.2 [30]. For each dose level, the NPS (Fig. 1A) and TTF (Fig. 1B) were computed on all the data from the ten acquisitions.

#### Noise power spectrum (NPS)

The NPS was computed using eight square regions of interest (ROI) of 222 × 222 pixels placed on 500 consecutive axial slices (50 slices for each of the 10 acquisitions) for PCCT and 400 consecutive axial slices (40 slices for each of the 10 acquisitions) as follows (Eq. 1):

1 $${{\mathrm{NPS}}}_{2{{\rm{D}}}}\left({f}_{x},{f}_{y}\right)=\frac{{\Delta }_{x}{\Delta }_{y}}{{L}_{x}{L}_{y}}\frac{1}{{N}_{{\mathrm{ROI}}}}\sum_{i=1}^{{N}_{{\mathrm{ROI}}}}{\left|{{\mathrm{FFT}}}_{2{{\rm{D}}}}\left\{{{\mathrm{ROI}}}_{i}\left(x,y\right)-{{\mathrm{FIT}}}_{i}(x,y)\right\}\right|}^{2}$$

wherein *Δ*_*x*_ and *Δ*_*y*_ are the pixel size in the *x*- and y-directions, respectively; FFT is the Fast Fourier Transform; *L*_*x*_ and *L*_*y*_ are the lengths of the ROIs in the *x*- and *y*-directions; *N*_ROI_ is the number of ROIs; ${{\mathrm{ROI}}}_{i}\left({{\rm{x}}},{{\rm{y}}}\right)$ is the mean pixel value of a ROI measured at the position (*x*,*y*) and ${{\mathrm{FIT}}}_{i}({{\rm{x}}},{{\rm{y}}})$ is a 2nd order polynomial fit of ${{\mathrm{ROI}}}_{i}\left({{\rm{x}}},{{\rm{y}}}\right)$. The raw data NPS1D curves were fitted using an 11th-order polynomial.

To assess noise magnitude, the square root of the area under the NPS2D curve was used, and the average spatial frequency of the NPS1D curve, (*f*_av_), was used for noise texture [31].

#### Task-based transfer function (TTF)

To assess spatial resolution, the TTF was computed using the circular edge technique [32] with the same number of slices on each CT system as for the NPS. TTF was calculated on air and on iodine at 10 mg/mL inserts. TTF values at 50% (*f*_50_) were used to assess spatial resolution.

#### Detectability index (*d*’)

The *d*’ of three clinical tasks was computed using a non-pre-whitening model observer with an eye filter according to the following equation (Eq. 2) [30, 31].

2 $${{d}^{{\prime} }}_{{\mathrm{NPWE}}}^{2}=\frac{{\left[\iint {{|W}\left(u,v\right)|}^{2}\times {{\mathrm{TTF}}}{\left(u,v\right)}^{2}\times E{\left(u,v\right)}^{2}{dudv}\right]}^{2}}{\iint {|W\left(u,v\right)|}^{2}\times {{\mathrm{TTF}}}{\left(u,v\right)}^{2}\times {{\mathrm{NPS}}}(u,v)\times E{(u,v)}^{4}{dudv}}$$

wherein u and v are the spatial frequencies in the *x*- and *y*-directions, E the eye filter that models the human visual system sensitivity to different spatial frequencies, and $W\left(u,v\right)$ is the task function defined as (Eq. 3):

3 $$W=\left|{{FFT}}_{2D}\left\{{h}_{1}\left(x,y\right)-{h}_{0}\left(x,y\right)\right\}\right|$$

wherein ${\mathrm{FFT}}$ is the Fast Fourier Transform, ${h}_{1}\left(x,y\right)$ and ${h}_{0}\left(x,y\right)$ are the object present and object absent hypotheses, respectively [30, 31, 33].

Three 5 mm-diameter nodules were defined to represent the three nodules of 100, −630, and −800 HU available in the anthropomorphic phantom. To account for the contrast between the three nodules and the lung of the phantom (−900 HU), lesions were simulated (as differences) with the following HU values: 1000 HU, 270 HU, and 100 HU. The TTF outcomes of the air insert were used for the first nodule, and the outcomes of the iodine insert for the other two.

The shape signal was circular, and the “Designer” contrast profile was used for all the simulated lesions [30, 31]. The interpretation conditions for calculating all *d*’ indexes were a 180-mm display size, a 500-mm viewing distance, and the Eckstein visual function [34].

### Subjective assessment of nodule detection

Five radiologists (from 3 to 25 years of experience) independently viewed the images containing three pulmonary nodules embedded in an anthropomorphic phantom. They were blinded to the nodule type, CT system, reconstruction algorithm, and dose level. The radiologists rated their confidence in detecting each nodule on a 5-point scale: 1 = very low, 2 = low, 3 = moderate, 4 = high, and 5 = excellent. Scores below 3 were considered insufficient for clinical use. For each nodule, the mean and standard deviation of the scores assigned by the five radiologists were subsequently calculated.

In addition, the radiologists subjectively assessed image noise and image smoothing (image appears blurred, blotchy, plastic-looking or unnatural) around each nodule using a five-point Likert scale: 1 = unacceptable (very strong image noise or image smoothing), 2 = suboptimal (strong image noise or image smoothing), 3 = acceptable (acceptable image noise or image smoothing), 4 = above average (low image noise or image smoothing), and 5 = excellent (very low or no image noise or image smoothing).

### Statistical analysis

Statistical analysis was performed using Biostatgv (https://marne.u707.jussieu.fr/biostatgv). For each subjective criterion, scores from the three nodules evaluated by the five radiologists were compared across dose levels within each CT system (PCCT, UHR-CT DLR, and UHR-CT IR), as well as across reconstruction algorithms at the same dose level for UHR-CT. Comparisons were made using the paired Mann–Whitney *U*-test, and a *p*-value < 0.05 was considered statistically significant.

## Results

### NPS

**Fig. 2:**
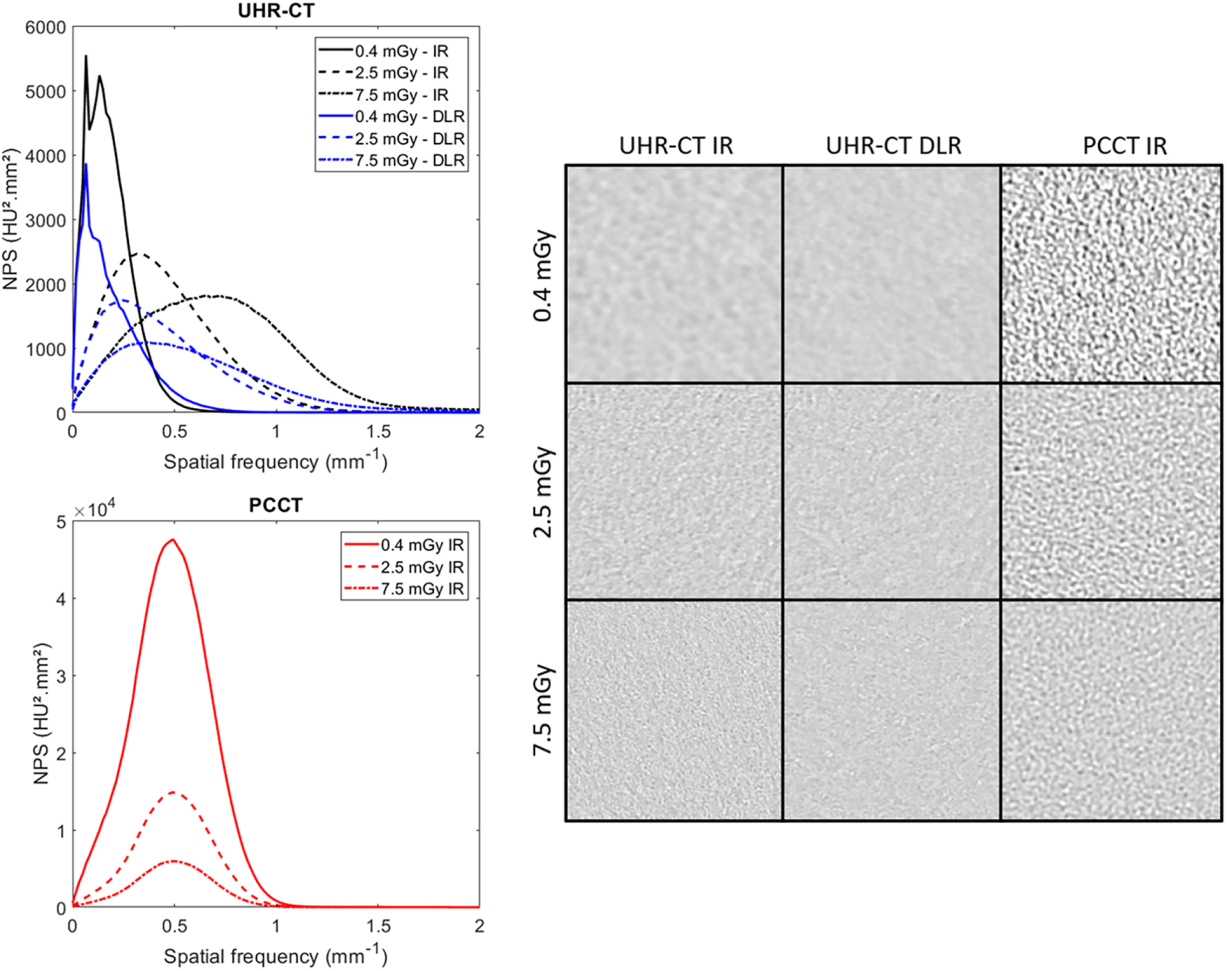
NPS curves and the 180 × 180 pixels square ROI images (window level: 600 HU and window width: −1600 HU) centered on the homogeneous section of the phantom obtained for the two CT systems, and at all dose levels

For PCCT, the amplitude of the NPS1D curves decreased as the dose level increased (Fig. 2 and Table 1). For all dose levels, the shape of the NPS1D curves remained similar, with NPS peaks occurring at comparable spatial frequencies (*f*_peak_ 0.48 ± 0.01 mm^−1^). The *f*_av_ values were similar according to the dose level (0.49 ± 0.01 mm^−1^).

For UHR-CT and both reconstruction algorithms, the transition from 7.5 mGy to 0.4 mGy increased the amplitude of the NPS1D curves (increase in NPS peak), and their distribution narrowed and concentrated on lower spatial frequencies (Fig. 2). This resulted in noise magnitude, *f*_peak_, and *f*_av_ values that decreased as the dose level decreased (Table 2). For all dose levels, the noise magnitude values were lower with DLR than with IR (−16.8% at 0.4 mGy, −18.0% at 2.5 mGy, and −33.4% at 7.5 mGy). For all dose levels, *f*_peak_ and *f*_av_ values were lower with DLR than with IR. The highest differences in *f*_peak_ and *f*_av_ values between DLR and IR were found at 7.5 mGy (−26.6 ± 2.5% at 0.4 and 2.5 mGy and −43.9% at 7.5 mGy for *f*_peak_ values and −6.0 ± 2.2% and −16.9% for *f*_av_ values, respectively).

**Table 2:** Values of noise magnitude, spatial frequency of the NPS peak (*f*_peak_), and average NPS spatial frequency (*f*_av_) were obtained for each dose level, the two systems, and the two algorithms for UHR-CT

	CTDI_vol_ (mGy)	UHR-CT IR	UHR-CT DLR	PCCT-IR
Noise magnitude (HU)	0.4	39.3	32.7	262.6
	2.5	66.0	54.1	146.5
	7.5	88.0	58.6	92.8
*f*_peak_ (mm^−1^)	0.4	0.116	0.083	0.478
	2.5	0.330	0.248	0.478
	7.5	0.677	0.380	0.494
*f*_av_ (mm^−1^)	0.4	0.198	0.183	0.485
	2.5	0.454	0.434	0.496
	7.5	0.720	0.598	0.498

*CT* computed tomography, *CTDI*_vol_ volume CT dose index, *DLR* deep-learning image reconstruction, *IR* iterative reconstruction, *PCCT* photon-counting CT, *UHR* ultra-high resolution

For all dose levels, noise magnitude values were higher with PCCT than with UHR-CT for both reconstruction algorithms. The differences in noise magnitude values between PCCT and UHR-CT decreased as the dose level increased (from 703.1% at 0.4 mGy to 58.4% at 7.5 mGy for UHR-CT DLR). For both reconstruction algorithms, *f*_av_ values were lower with UHR-CT than with PCCT at 0.4 and 2.5 mGy, whereas the opposite trend was observed at 7.5 mGy.

### TTF

**Fig. 3:**
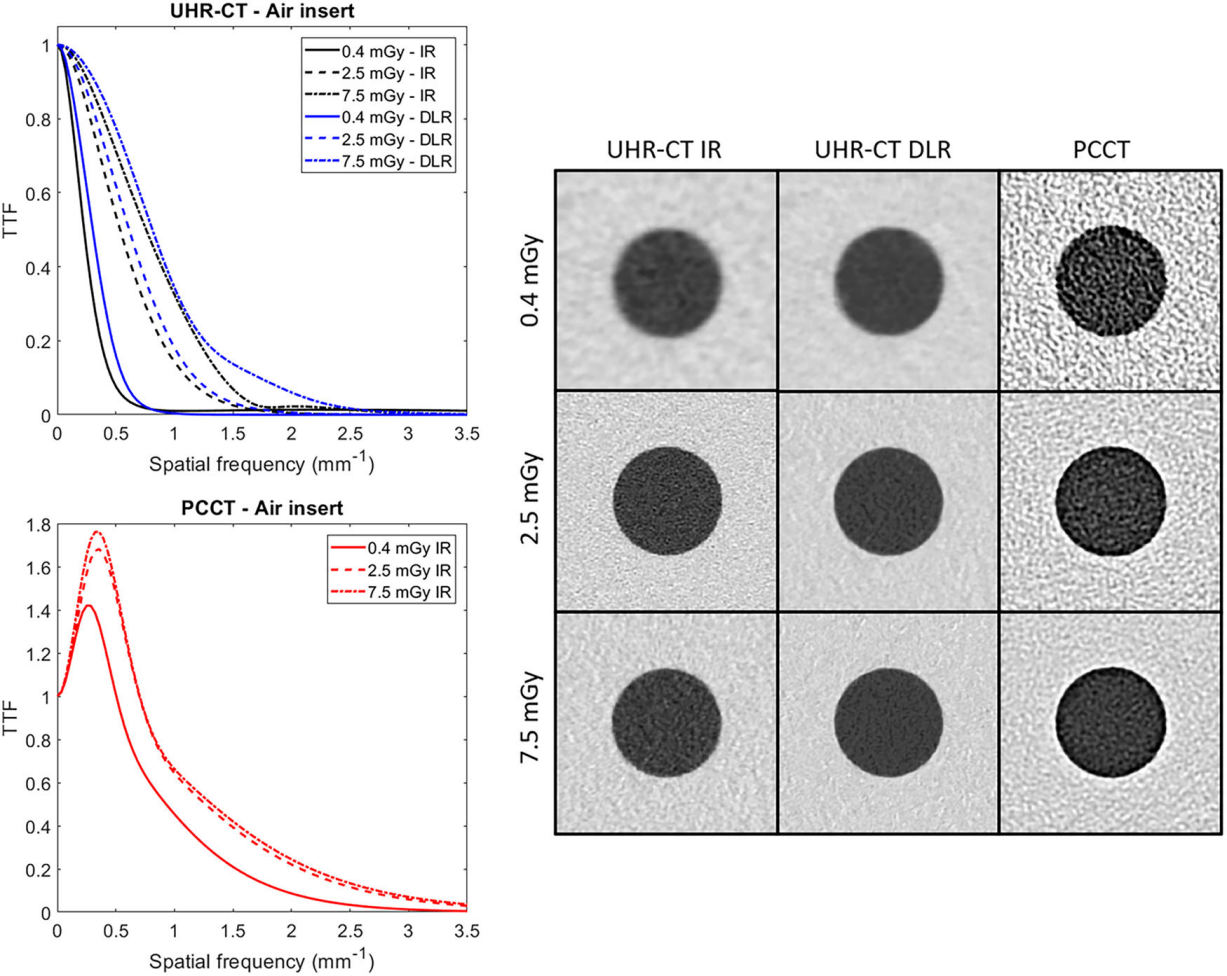
TTF curves for the air insert, and the 180 × 180 pixels square ROI images (window level: 600 HU and window width: −1600 HU) centered on the air insert obtained for the two CT systems, and all dose levels

**Fig. 4:**
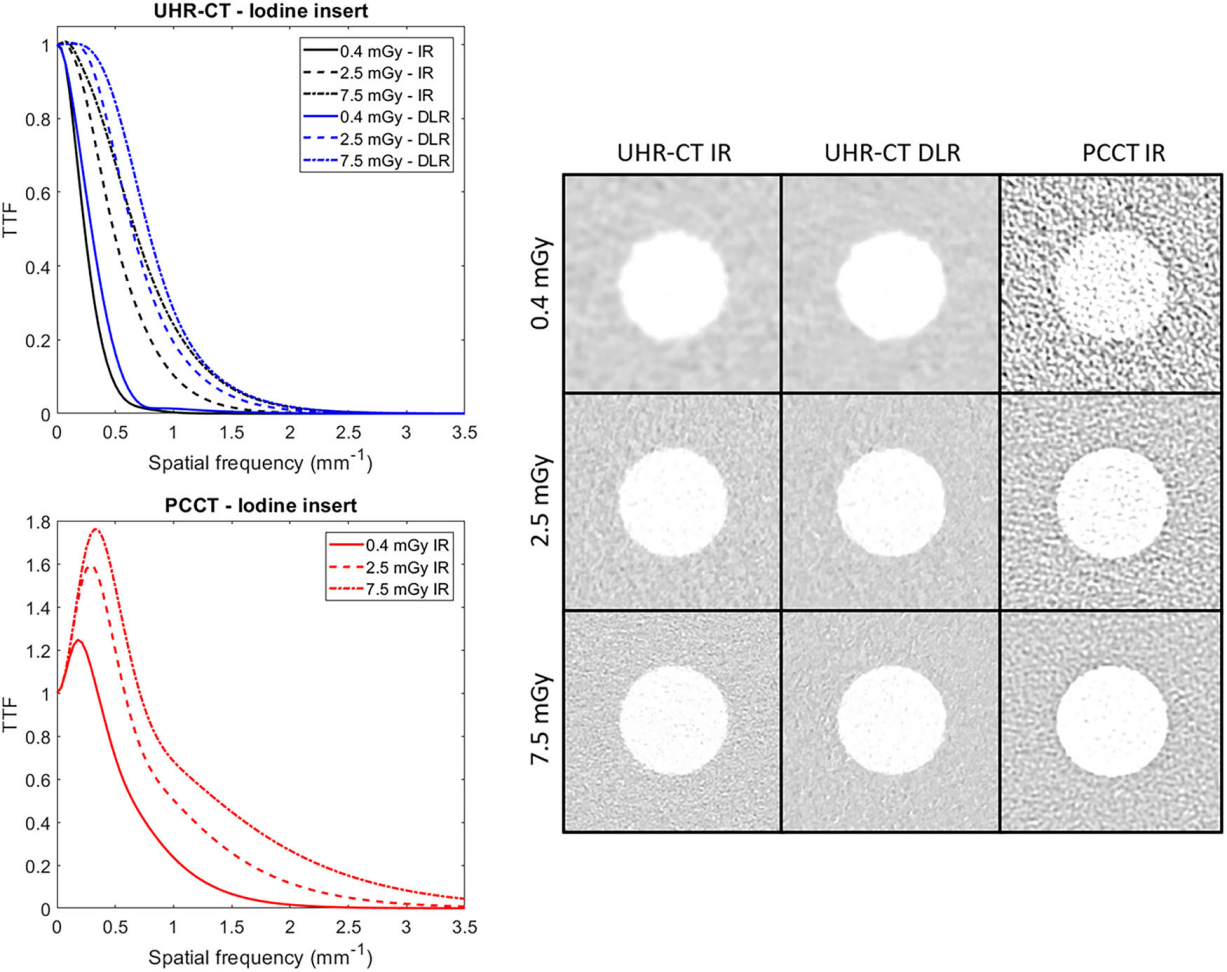
TTF curves for the iodine insert, and the 180 × 180 pixels square ROI images (window level: 600 HU and window width: −1600 HU) centered on the iodine insert obtained for the two CT systems, and at all dose levels

For the PCCT and the two inserts, the TTF curves and *f*_50_ values shifted towards higher frequencies as the dose increased (Figs. 3 and 4 and Table 3). The TTF curves also exhibited an overshoot at low frequencies, and their height increased with the dose. *f*_50_ values were higher with air insert than with the iodine insert at 0.4 mGy (42.9%) and 2.5 mGy (25.8%), and had approximately the same magnitude at 7.5 mGy (−4.1%).

**Table 3:** TTF values at 50% (*f*_50_) for air and iodine at 10 mg/mL inserts obtained for each dose level, the two systems, and the two algorithms for UHR-CT

	CTDI_vol_ (mGy)	UHR-IR	UHR-DLR	PCCT-IR
*f*_50_ (mm^−^^1^) Air insert	0.4	0.229	0.303	0.929
	2.5	0.536	0.614	1.264
	7.5	0.758	0.812	1.320
*f*_50_ (mm^−^^1^) Iodine insert at 10 mg/mL	0.4	0.237	0.296	0.650
	2.5	0.483	0.645	1.005
	7.5	0.668	0.782	1.377

*CT* computed tomography, *CTDI*_vol_ volume CT dose index, *DLR* deep-learning image reconstruction, *IR* iterative reconstruction, *PCCT* photon-counting CT, *UHR* ultra-high resolution

For UHR-CT, the two inserts and both reconstruction algorithms, the TTF curves and *f*_50_ values shifted towards higher frequencies as the dose increased (Figs. 3 and 4 and Table 2). For both inserts and all dose levels, *f*_50_ values were higher with DLR than with IR algorithms. For the iodine insert and at all dose levels, the mean difference between DLR and IR was 25.2 ± 8.2%. For air inserts, the difference between DLR and IR decreased as the dose level increased (from 32.3% at 0.4 mGy to 7.1% at 7.5 mGy).

For both inserts, *f*_50_ values were higher with PCCT than with UHR-CT for both algorithms, and the differences between them decreased as the dose level increased.

### Detectability indexes

**Fig. 5:**
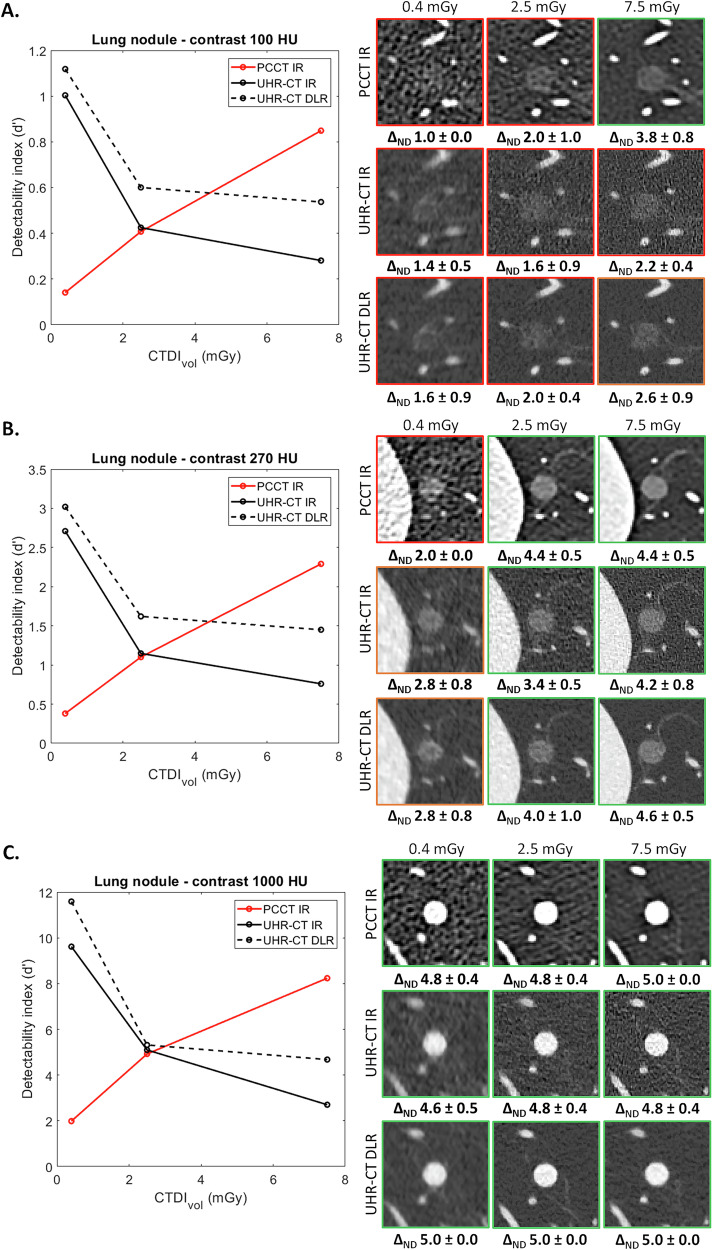
Curves of detectability index values and average confidence scores for nodule detection (Δ_ND_) obtained by the five radiologists on the 80 × 80 pixels square ROI images (window level: 600 HU and window width: −1600 HU) centered on the three nodules with contrast values of 100 HU (**A**), 270 HU (**B**), and 1000 HU (**C**) obtained for each dose level, the two systems and the two algorithms for UHR-CT

For PCCT, the *d*’ values of the three simulated chest nodules increased as the dose level increased (Fig. 5). For UHR-CT and the two reconstruction algorithms, the opposite was found.

For UHR-CT, *d*’ values were higher with DLR than with IR for all simulated chest nodules at all dose levels. Differences between them increased as the dose level increased, except for the lung nodule at 1000 HU, where the *d*’ values were close for both algorithms at 2.5 mGy.

For all simulated chest nodules, the highest *d*’ values were found with PCCT at 7.5 mGy and with UHR-CT-DLR at 2.5 and 0.4 mGy. At 2.5 mGy, similar *d*’ values were found for PCCT and UHR-CT-IR for all simulated chest nodules, and the lowest *d*’ values were found with PCCT at 0.4 mGy.

### Subjective assessment of image noise and image smoothing

Table 4 depicts the noise and smoothing scores rated by the five radiologists for each of the chest nodule images. For PCCT, the global image noise score increased significantly as the dose level increased (*p* < 0.05) and was rated as unacceptable or suboptimal at 0.4 mGy. For each dose level, the global image noise score obtained with DLR in UHR-CT was significantly higher (rated as less noisy) than with IR (*p* < 0.05), For UHR-CT DLR, global image noise scores were similar across all dose levels (*p* > 0.05), ranging from “acceptable” to “above average.” A similar trend was observed for UHR-CT IR (*p* > 0.05), but scores ranged from “acceptable” to “suboptimal”.

**Table 4:** Mean (±standard deviation) image noise and image texture scores averaged across the three nodules, as rated by the five radiologists (R1–R5), for each dose level, for both CT systems, and for both reconstruction algorithms in UHR-CT

Criteria	CTDI_vol_	CT systems	R1	R2	R3	R4	R5	Global score
Image noise	0.4 mGy	PCCT-IR	1.7 ± 1.2	1.0 ± 0.0	1.0 ± 0.0	1.7 ± 1.2	1.0 ± 0.0	1.3 ± 0.7
		UHR-CT IR	1.7 ± 0.6	2.3 ± 0.6	2.7 ± 0.6	3.0 ± 0.0	3.3 ± 0.6	2.6 ± 0.7
		UHR-CT DLR	3.7 ± 0.6	3.7 ± 0.6	4.0 ± 0.0	3.7 ± 0.6	3.3 ± 0.6	3.7 ± 0.5
	2.5 mGy	PCCT-IR	2.0 ± 0.0	2.7 ± 1.2	2.0 ± 0.0	3.0 ± 1.7	2.3 ± 1.2	2.4 ± 1.0
		UHR-CT IR	2.0 ± 0.0	2.0 ± 0.0	2.3 ± 0.6	2.7 ± 0.6	1.7 ± 0.6	2.1 ± 0.5
		UHR-CT DLR	3.3 ± 0.6	4.0 ± 1.0	3.7 ± 1.5	4.0 ± 0.0	3.3 ± 0.6	3.7 ± 0.8
	7.5 mGy	PCCT-IR	3.0 ± 0.0	4.0 ± 0.0	4.0 ± 1.0	4.7 ± 0.6	3.3 ± 0.6	3.8 ± 0.8
		UHR-CT IR	1.7 ± 0.6	2.0 ± 0.0	3.0 ± 1.0	2.3 ± 0.6	1.7 ± 0.6	2.1 ± 0.7
		UHR-CT DLR	4.0 ± 0.0	5.0 ± 0.0	4.7 ± 0.6	4.7 ± 0.6	3.3 ± 0.6	4.3 ± 0.7
Image smoothing	0.4 mGy	PCCT-IR	4.3 ± 0.6	5.0 ± 0.0	4.3 ± 1.2	4.0 ± 0.0	5.0 ± 0.0	4.5 ± 0.6
		UHR-CT IR	2.0 ± 0.0	1.7 ± 0.6	2.3 ± 0.6	2.3 ± 0.6	2.7 ± 0.6	2.1 ± 0.7
		UHR-CT DLR	1.3 ± 0.6	2.0 ± 1.0	2.3 ± 0.6	3.0 ± 0.0	2.7 ± 0.6	2.3 ± 0.6
	2.5 mGy	PCCT-IR	4.7 ± 0.6	3.3 ± 0.6	3.7 ± 0.6	3.3 ± 0.6	4.0 ± 1.0	3.8 ± 0.8
		UHR-CT IR	2.7 ± 1.2	3.0 ± 1.0	2.3 ± 0.6	3.3 ± 0.6	4.3 ± 0.6	3.1 ± 1.0
		UHR-CT DLR	2.3 ± 0.6	2.0 ± 0.0	2.3 ± 0.6	3.0 ± 1.0	3.0 ± 0.0	2.5 ± 0.6
	7.5 mGy	PCCT-IR	3.7 ± 0.6	2.7 ± 0.6	3.0 ± 1.0	3.0 ± 0.0	2.7 ± 0.6	3.0 ± 0.7
		UHR-CT IR	3.0 ± 1.0	4.0 ± 0.0	2.3 ± 0.6	4.3 ± 0.6	4.3 ± 0.6	3.6 ± 1.0
		UHR-CT DLR	2.7 ± 1.2	2.0 ± 0.0	2.0 ± 0.0	4.0 ± 0.0	3.0 ± 0.0	2.7 ± 0.9

*CT* computed tomography, *CTDI*_vol_ volume CT dose index, *DLR* deep-learning image reconstruction, *IR* iterative reconstruction, *PCCT* photon-counting CT, *SD* standard deviation, *UHR* ultra-high resolution

For PCCT, the global image smoothing score was significantly decreased (rated as smoother) with increasing dose level (*p* < 0.05). Average global scores ranged from above average to excellent at 0.4 mGy and were acceptable at 7.5 mGy. In contrast, for UHR-CT, both reconstruction algorithms showed more pronounced image smoothing with decreasing dose level. Global average score increased significantly as the dose level increased for IR (*p* < 0.05) but not significantly for DLR (*p* > 0.05). At 7.5 mGy,  the global average score was significantly higher with IR than with DLR (*p* < 0.05), and the differences between them were not significant at other dose levels (*p* > 0.05).

### Subjective assessment of nodule detection

Table 5 depicts the confidence level score obtained by each of the 5 radiologists for the detection of each nodule. For each nodule, the mean score increased significantly as the dose level increased for PCCT and UHR-CT for both algorithms (*p* < 0.05). For UHR-CT and all nodules, the mean score was higher with DLR than with IR, but not significantly for each dose level (*p* > 0.05) (Fig. 5).

**Table 5:** Confidence level score for the detection of each nodule obtained by each of the 5 radiologists (R1 to R5), for each dose level, the two systems, and the two algorithms for UHR-CT

Nodule contrast	CTDI_vol_ (mGy)	CT systems	R1	R2	R3	R4	R5	Mean ± SD score
100 HU	0.4 mGy	PCCT-IR	**1**	**1**	**1**	**1**	**1**	1.0 ± 0.0
		UHR-CT IR	**1**	**2**	**2**	**1**	**1**	1.4 ± 0.5
		UHR-CT DLR	**1**	**2**	3	**1**	**1**	1.6 ± 0.9
	2.5 mGy	PCCT-IR	**1**	3	3	**1**	**2**	2.0 ± 1.0
		UHR-CT IR	**1**	**2**	3	**1**	**1**	1.6 ± 0.9
		UHR-CT DLR	**2**	3	**2**	**2**	**2**	2.0 ± 0.4
	7.5 mGy	PCCT-IR	4	5	4	3	3	3.8 ± 0.8
		UHR-CT IR	**2**	**2**	3	**2**	**2**	2.2 ± 0.4
		UHR-CT DLR	**2**	4	3	**2**	**2**	2.6 ± 0.9
270 HU	0.4 mGy	PCCT-IR	**2**	**2**	**2**	**2**	**2**	2.0 ± 0.0
		UHR-CT IR	**2**	4	3	**2**	3	2.8 ± 0.8
		UHR-CT DLR	**2**	4	3	**2**	3	2.8 ± 0.8
	2.5 mGy	PCCT-IR	4	5	5	4	4	4.4 ± 0.5
		UHR-CT IR	3	4	4	3	3	3.4 ± 0.5
		UHR-CT DLR	4	5	5	3	3	4.0 ± 1.0
	7.5 mGy	PCCT-IR	4	5	5	4	4	4.4 ± 0.5
		UHR-CT IR	4	5	5	3	4	4.2 ± 0.8
		UHR-CT DLR	5	5	5	4	4	4.6 ± 0.5
1000 HU	0.4 mGy	PCCT-IR	5	5	4	5	5	4.8 ± 0.4
		UHR-CT IR	5	4	4	5	5	4.6 ± 0.5
		UHR-CT DLR	5	5	5	5	5	5.0 ± 0.0
	2.5 mGy	PCCT-IR	5	5	4	5	5	4.8 ± 0.4
		UHR-CT IR	5	5	4	5	5	4.8 ± 0.4
		UHR-CT DLR	5	5	5	5	5	5.0 ± 0.0
	7.5 mGy	PCCT-IR	5	5	5	5	5	5.0 ± 0.0
		UHR-CT IR	5	5	4	5	5	4.8 ± 0.4
		UHR-CT DLR	5	5	5	5	5	5.0 ± 0.0

*CT* computed tomography, *CTDI*_vol_ volume CT dose index, *DLR* deep-learning image reconstruction, *IR* iterative reconstruction, *PCCT* photon-counting CT, *SD* standard deviation, *UHR* ultra-high resolution

Score below 3 (in bold) were considered insufficient for clinical use

For the nodule with a contrast of 100 HU (Fig. 5A), the mean scores were below 3 for all CT systems at all dose levels (highlighted in red), except for PCCT at 7.5 mGy (3.8 ± 0.8), highlighted in green. For this dose level, 2 out of 5 radiologists assigned a score ≥ 3 for UHR-CT-DLR (highlighted in orange).

For the nodule with a contrast of 270 HU (Fig. 5B), the mean scores were ≥ 3 for all CT systems at 2.5 and 7.5 mGy. At 0.4 mGy, the mean scores were below 3 for all CT systems, but for UHR-CT and both algorithms, 3 out of 5 radiologists assigned a score ≥ 3 (highlighted in orange).

For the nodule with a contrast of 1000 HU (Fig. 5.C), scores were ≥ 4 (high or excellent) for all CT systems and all dose levels.

## Discussion

In this study, we assessed the quality of chest CT images under UHR conditions in routine clinical practice using the only commercially available UHR-CT equipped with EIDs, and the UHR mode of the only wide-bore PCCT available. To do this, a task-based image quality assessment was performed to evaluate noise magnitude, noise texture, and spatial resolution. The detectability of three types of small pulmonary nodules with different contrast values was evaluated using the detectability index, and the confidence in detecting these nodules was assessed by five radiologists. The results of this study showed that the choice of reconstruction kernel and algorithm had different impacts on noise magnitude, noise texture, spatial resolution, detectability, and confidence in nodule detection.

The NPS results obtained with PCCT according to dose level were similar to those reported in previous studies [13, 24]. Noise magnitude increased as the dose decreased, whereas noise texture remained similar, a behavior confirmed visually by similar image texture with increasing image noise at lower dose levels. Higher noise magnitude and *f*_av_ values than those reported in the literature were observed, probably due to the use of an extended matrix, thinner reconstruction slices made possible by the UHR mode, and a lower QIR level [13, 24]. The subjective assessment of image noise around the nodules studied, performed by the five radiologists, confirmed the objective NPS results, with perceived image noise increasing as the dose decreased. Regarding image smoothing, scores ranged from acceptable to excellent across all dose levels, with higher scores observed at lower doses. This counterintuitive outcome at low doses may be explained by the higher noise level, which impairs the radiologists’ ability to perceive image smoothness and thus makes it difficult to evaluate. In contrast, for UHR-CT, the frequency distribution of noise varied with dose level. For both reconstruction algorithms, the NPS curves gradually grouped together and shifted towards lower spatial frequencies as the dose decreased, whereas the amplitude increased. This resulted in reduced noise magnitude, *f*_av_ and *f*_peak_ values from 7.5 to 0.4 mGy. Furthermore, the DLR algorithm reduced noise magnitude and shifted the NPS curves towards lower frequencies than with IR at all dose levels. Visually, these results corresponded to smoother, less noisy images from 7.5 to 0.4 mGy for both algorithms and with DLR, compared to IR at all dose levels. The increase in image smoothing with decreasing dose was confirmed by subjective analysis performed by the radiologists. For IR, image smoothing was rated between acceptable and suboptimal at 0.4 mGy, whereas for DLR, scores were maintained across all dose levels. Furthermore, image noise scores were relatively consistent across dose levels for each reconstruction algorithm. Finally, noise magnitude values were higher at all dose levels with PCCT than with UHR-CT, but the image texture (*f*_av_ and *f*_peak_ values) was less smooth with PCCT, except at 7.5 mGy.

The TTF results show that the curves shifted towards lower spatial frequencies as the dose level decreased for both CT systems and both inserts. The impact of dose level was more pronounced for UHR-CT with both algorithms than for PCCT, in particular for air insert. Furthermore, for each CT system and both algorithms for UHR-CT, the *f*_50_ values differed depending on the insert used. These results confirmed that the reconstruction algorithms used on both CT systems had non-linear properties, resulting in spatial resolution that depended on the dose level and the contrast insert used. In addition, for PCCT, the TTF curves showed an overshoot placed at low spatial frequencies that was not, present in the UHR-CT curves. These overshoots, as previously reported [13, 24], were directly related to the use of edge enhancement with this type of kernel (Bl60). Finally, for all dose levels and both inserts, *f*_50_ values were higher for PCCT than for UHR-CT and higher for DLR than for IR with UHR-CT.

The differences in NPS and TTF results observed between the two CT systems were related to differences in the reconstruction algorithms and kernels. For PCCT, the NPS results indicate that the kernel behaved consistently across dose levels, with a probably unchanged cut-off frequency. In contrast, for UHR-CT, the kernels associated with the two reconstruction algorithms exhibited dose-dependent behavior. The results suggest a probable shift of the cut-off frequency towards lower spatial frequencies as the dose decreased. A lower cut-off frequency emphasizes low-frequency information (global image content and useful signal) at the expense of high-frequency components (image noise and spatial resolution). This behavior might be used to reduce image noise at the expense of image texture (e.g., image smoothing) and spatial resolution. Overall, the results suggest that the cutoff frequency used by the PCCT kernel was higher than the cutoff frequency used by the other two kernels with UHR-CT.

These reconstruction characteristics also influenced the detectability of simulated pulmonary nodules. For both CT systems, the *d*’ values increased with lesion contrast. For PCCT, the *d*’ values increased as the dose level increased, in agreement with previously published results [24]. Conversely, with UHR-CT, the opposite was found for both algorithms. This unusual behavior was directly related to variations in noise magnitude depending on the dose level, but also to the operation of the kernels specified above. We also found that *d*’ values were higher with DLR than with IR for all simulated nodules, which is also consistent with results published for other kernels [35]. Based on all these results, the highest *d*’ values for PCCT were found at 7.5 mGy and for UHR-CT DLR at 0.4 and 2.5 mGy.

The subjective assessment made by the five radiologists showed substantial variability. The mean confidence score of radiologists in detecting nodules increased with dose level, nodule contrast, and with DLR compared to IR for UHR-CT. These trends were consistent with *d*’ values for PCCT. However, for UHR-CT, the dose-dependent variation in subjective scores was the opposite of what was observed for *d*’. In particular, higher *d*’ values for UHR-CT at 0.4 mGy did not translate into clinically sufficient detection confidence for nodules with contrasts of 100 HU and 270 HU. These findings confirm that *d*’ alone may overestimate clinical performance and must be complemented by a subjective assessment.

Finally, the combined results of the objective and subjective assessments showed that the detectability of simulated small pulmonary nodules and the confidence in their detection depended both on lesion contrast and dose level. Under UHR conditions, diagnostic confidence was clinically satisfactory for high-contrast solid nodules (1000 HU) at LD and ULD levels and for subsolid nodules (270 HU) at LD. Furthermore, confidence in the detection of low-contrast nodules (100 HU) was insufficient for clinical use under UHR conditions for both systems, except for PCCT at 7.5 mGy. This limitation suggests that, to improve the detection of low-contrast nodules at low or ULDs, an additional reconstruction using a standard matrix and/or greater slice thickness may be beneficial. At the same time, the routine clinical implementation of UHR acquisitions using an extended matrix and ultra-thin slice thickness raises practical concerns. Compared with standard CT protocols, these parameters substantially increase data volume and may lead to a higher reading workload, potentially limiting their use in screening or follow-up settings. In this context, ULD acquisitions, recommended for lung cancer screening [7, 11–13], could still be used under UHR conditions to detect solid or subsolid nodules, combined with these additional standard reconstructions for detecting low-contrast nodules. This combined approach may offer a pragmatic balance between detectability performance and clinical feasibility. These promising preliminary results on phantoms must now be confirmed on a cohort of patients undergoing chest CT examinations.

This study has certain limitations. First, acquisitions were made on two static phantoms of different sizes, and these do not represent the full range of patient morphologies. In addition, simulated nodules may not fully replicate the complexity of anatomical structures. Different results might have been obtained with nodules of different sizes and contrasts positioned at different locations in the phantom. Furthermore, phantom-based detectability does not adequately capture the effects of complex lung anatomy, motion, vessel adjacency, and heterogeneous nodule appearance. In addition, only one nodule per attenuation level was evaluated in the subjective analysis, which may limit the generalizability of findings and does not account for inter-nodule variability. Evaluating a larger number of nodules per attenuation level may have provided a more robust assessment of subjective detectability. Confidence in nodules detection was also assessed subjectively using a 5-point confidence scale, which may not directly reflect actual detection performance. In any case, this subjective assessment method has inherent limitations that restrict the clinical applicability of results. Furthermore, only one reconstruction kernel and a single IR/DLR level were evaluated under UHR conditions. The choice of these parameters was made to compare typical clinical configurations rather than optimized or equivalent reconstruction conditions. Different settings may have yielded different results. Finally, a fixed CTDI_vol_ was used rather than tube current modulation, which may also have influenced the results.

In conclusion, this preliminary study demonstrates that, under UHR conditions, image quality and pulmonary nodule detectability strongly depend on reconstruction algorithms and kernels. PCCT showed dose-invariant noise texture and higher effective spatial resolution, whereas UHR-CT relied on dose-dependent image noise suppression, leading to smoother images and reduced spatial frequency. High-contrast nodules can be detected with UHR chest CT imaging at low- and ULD, but optimized reconstruction strategies to ensure reliable detection of low-contrast lesions in clinical practice are still required. These preclinical and task-specific results, while encouraging, are exploratory and limited to two phantoms. Further validation in clinical cohorts will be essential to supplement this preliminary study.

## Abbreviations

CT: Computed tomography
CTDI_vol_: Volume CT dose index
*d*’: Detectability index
DLR: Deep-learning image reconstruction algorithm
EID: Energy-integrating detectors
HU: Hounsfield unit
IR: Iterative reconstruction algorithm
LD: Low-dose
NPS: Noise power spectrum
PCCT: Photon-counting CT
PCD: Photon-counting detectors
TTF: Task-based transfer function
UHR: Ultra-high resolution
ULD: Ultra-low dose
